# HOXA10 Regulates the Synthesis of Cholesterol in Endometrial Stromal Cells

**DOI:** 10.3389/fendo.2022.852671

**Published:** 2022-04-25

**Authors:** Meixing Yu, Jia Tang, Yanqing Huang, Chenbing Guo, Peng Du, Ning Li, Qingli Quan

**Affiliations:** ^1^ Guangzhou Women and Children’s Medical Center, Guangzhou Medical University, Guangzhou, China; ^2^ NHC Key Laboratory of Male Reproduction and Genetics, Guangdong Provincial Reproductive Science Institute (Guangdong Provincial Fertility Hospital), Guangzhou, China

**Keywords:** ovarian endometriosis, cholesterol synthesis, HOXA10 gene, endometrial stromal cell, estrogen

## Abstract

**Background:**

The expression of homeobox A10 (HOXA10) in endometrial stromal cells is regulated by steroid hormones, especially by estrogen. As a precursor molecule of estrogen, abnormal cholesterol metabolism is significantly positively correlated with endometriosis. The purpose of this study was to explore the regulation of HOXA10 on cholesterol synthesis in endometrial stromal cells.

**Method:**

mRNA expression data of eutopic endometrial stromal cell (ESC) and ovarian endometriotic cysts stromal cell (OESC) were download from the Gene Expression Omnibus (GEO) databases. Overexpression and silence of HOXA10 were conducted in cultured ESC and subjected to mRNA sequencing. The differentially expressed genes (DEGs) were selected by analyzing the sequencing data. Weighted gene co-expression network analysis (WGCNA) was applied to identify the key genes associated with HOXA10. The methylation rate of HOXA10 CpGs and the correlation between HOXA10 expression and the methylation in eutopic endometrial tissue (EU) and ovarian cyst (OC) were analyzed.

**Results:**

HOXA10 in ESC was significantly higher expressed than that in OESC. Six key genes (HMGCR, MSMO1, ACAT2, HMGCS1, EBP, and SQLE), which were regulated by HOXA10, were identified from the salmon4 module by WGCNA. All these key genes were enriched in cholesterol synthesis. Moreover, the expression of HOXA10 was negatively related to its CpGs methylation rate.

**Conclusion:**

In this study, six key genes that were regulated by HOXA10 were selected, and all of them were enriched in cholesterol synthesis. This finding provided a new insight into the metabolic mechanism of cholesterol in ESC. It also provided a potential treatment strategy for cholesterol metabolism maladjustment in patients with ovarian endometriosis.

## Introduction

Ovarian endometriosis (OEM) is a common gynecological disease and characterized by the presence of endometrial tissue outside the endometrial cavity, causing chronical pain and infertility (1–3). OEM is also an estrogen-dependent disease (4). Estrogen not only promotes the proliferation of normal endometrium and improves endometrial tissue implantation to the peritoneum but also stimulates local and systemic inflammation (5, 6).

One of the prerequisites for uterus normal function is the endometrium receptivity in which HOXA10, a transcription factor encoding gene, plays critical role (7, 8). In human endometrium, HOXA10 is expressed in both glandular and stromal cells and regulated by estrogens (9). HOXA10 is regulated by steroid hormones because there are functional estrogen response elements (EREs) in the 5′ transcription start site of HOXA10 (10). Additionally, HOXA10 also has a strong correlation with serum lipid in polycystic ovary syndrome (11).

It is reported that local steroid hormones (such as estrone, estradiol, and progesterone) of endometrial and endometriotic tissues are more determined by active local synthesis and metabolism instead of being determined by passive diffusion from circulating steroids (12, 13). In endometriotic lesions, estradiol biosynthesis is higher and estradiol inactivation is lower compared with the normal endometrium (12, 14). Hence, abnormal steroid hormone biosynthesis and metabolism is one of the factors contributing to endometriosis.

The parent molecule of steroid hormone is cholesterol (15). Abnormal cholesterol metabolism has been observed to be correlated with endometriosis (16). Melo et al. reported that the serum levels of low-density lipoprotein (LDL), non-high-density lipoprotein (non-HDL), triglyceride (TG), and total cholesterol (TC) are higher, and even the HDL : TC ratio is lower in patients with endometriosis compared with those without endometriosis (16). Additionally, Mu et al. found that patients with confirmed endometriosis had a significant greater risk of cardiovascular disease than patients without endometriosis (17). Furthermore, gonadotropin-releasing hormone (GnRH) antagonist, as a common medicine used in endometriosis treatment, can inhibit ovarian follicular growth and ovulation, leading to a decreased production of circulating estradiol and a reduced growth of ectopic endometrium. As a side effect of GnRH antagonist, the risk of cardiovascular disease caused by increased serum lipid (TG, LDL, HDL, and even the ratio of LDL cholesterol to HDL cholesterol) also increases at the prolonged low estrogen state (4, 18). These indicate the important role of regulation of cholesterol synthesis in EMs.

Considering the irreplaceable role of HOXA10 in endometrium function and its potential role in cholesterol synthesis, we first analyzed the expression of HOXA10 in both ESC and OESC and performed HOXA10 overexpression and RNA interference (RNAi) on the ESC. Then, the key genes that were associated with HOXA10 expression were screened. Furthermore, given that an abnormal hypermethylation of HOXA10 has been found in ectopic lesions (19), we also detected the methylation rate of the representative CpG sites of HOXA10 in eutopic and ectopic tissue. We want to figure out how HOXA10 acts in cholesterol synthesis in the endometrium. Our research partly illustrated the role of HOXA10 in cholesterol synthesis in endometriosis, which provides new insights for the development and treatment for OEMs.

## Methods and Materials

### Patients Inclusion

Considering the potential regulatory effect of HOXA10 on the estrogen metabolism pathway, we first excluded patients who received hormone therapy. We enrolled 48 women with a history of OEMs or non-endometriosis-related disease (**Supplementary Table S1**). All study participants had a normal menstrual cycle and had not used oral contraception, hormonal therapy, or an intrauterine device for at least 3 months before the endometrial biopsy. Tissue samples were collected from patients by laparoscopic surgery or hysteroscopy during the proliferative phase of their menstrual cycle for the evaluation of pelvic pain, suspected ovarian cysts, or other unknown endometrial diseases. Then, all diagnoses were confirmed by tissue biopsy.

### Data Collection and the HOXA10 mRNA Expression Level Analysis in ESC and OESC

The mRNA expression microarray dataset (GSE136412) was downloaded from the GEO database (https://www.ncbi.nlm.nih.gov/geo/query/acc.cgi?acc). The mRNA expression of HOXA10 and the key genes of ESC and OESC were analyzed (**Supplementary Table S2**).

### ESC Isolation, Purification, and Identification

ESC used in our study were respectively isolated from five eutopic endometrium tissues from patients with non-endometriosis-related diseases. Tissues were digested with 1 mg/ml type IV collagenases (Solarbio, Beijing, China) and 150 U/ml DNase I (Tiangen Biotech, Beijing, China) in 37°C for 30 min and homogenized into single-cell suspension. Then, cells, which were filtered through a 40-μm filter (Solarbio, CHN), were collected and resuspended in Dulbecco’s modified Eagle’s medium (DMEM)/F12 complete medium (HyClone, Logan, UT, USA) containing 10% fetal bovine serum (FBS) (GIBCO, Grand Island, NY, USA). ESC with high purity were cultured by differential adherent and identified by using Alexa Flour 488 anti-human vimentin antibody (BD Biosciences, USA) and Alexa Flour 647 anti-human cytokeratin antibody (BD Biosciences, Franklin Lakes, NJ, USA). The flow cytometry data and gating strategies are provided in **Supplementary Data 1**.

### Over-Expression and Silence of HOXA10 in Cultured ESC

For HOXA10 over-expression, the pHBLV-ZsGreen-HOXA10 plasmid (Han Bio, Shanghai, China) and packaging plasmid (PSPAX2, pCMV-VSVG) (Han Bio, Shanghai, China) were transfected into 293T (Procell, Wuhan, China) to package the recombinant lentivirus. The plasmid pHBLV-ZsGreen was set as control. Virus was collected by ultracentrifugation, and the final concentration was 2×10^7^ virus/ml. ESC cells of HOXA10 over-expression group (HOXA10OE) and control group (ZsGreen) were treated with 0.8 μg/μl polybrene for 30 min, respectively. Then, ESC cells in both groups were infected with corresponding viruses, respectively [multiplicity of infection (MOI) ratio = 10]. For HOXA10 silence, ESC cells of HOXA10 silence group (siHOXA10) and control group (siNC) were transfected with 50 nM of HOXA10 siRNA (RIBOBIO, Guangzhou, China) and 50 nM of NC siRNA (RIBOBIO, Guangzhou, China), respectively, by Lipofectamine 3000 Transfection Reagent lipo3000 (Thermo Fisher Scientific, Waltham, MA, USA). Total mRNAs of all treatment groups and control groups were extracted with an RNeasy Mini kit (Qiagen, Hilden, Germary) and subjected to RNA sequencing (Berry Genomic, Illumina Nova 6000). The fragments per kilobase million (FPKM) of RNA-seq data are provided in **Supplementary Table S3**. The sequence of HOXA10 siRNA was 5′-GAGCTCACAGCCAACTTTA -3′.

### Differentially Expressed Genes Screening

Two groups of differentially expressed genes were screened respectively in both HOXA10-overexpresed ESC and HOXA10-RNAi ESC by comparing with their control group *via* the “limma” R package, according to the criteria of adj.P.Val <0.05 and |logFC| > 2 (20). By merging the two sets of different genes and removing duplicate genes, a set of genes was screened as DEGs.

### Weighted Gene Co-Expression Network Analysis

WGCNA is used to cluster highly coordinated gene sets and determine phenotype-related genes based on their correlation (21). To identify the related genes of HOXA10, we used WGCNA R package to analyze the mRNA expression data of HOXA10OE group, siHOXA10 group, and control groups. The soft-thresholding power was determined based on a scale-free R^2^ = 0.85. Similar dynamic modules were merged by setting 0.2 as the dissimilarity threshold. Pearson correlation analysis was used to selected the model related to HOXA10 expression. HOXA10-associated genes were identified when the gene significance (GS) is **>**0.8 and module membership (MM) is **>**0.8 in the selected model.

### PPI and Cystoscope

Protein–protein interactions (PPIs) are a vital mechanism for the regulation and coordination of most biological processes within the cell (22). Intersection genes were selected as candidate genes between the DEGs and HOXA10-associated genes screened by WGCNA. To investigate the key genes that were related to HOXA10, PPIs were constructed in the database (https://www.string-db.org) and visualized by Cytoscape software (version 3.2.1) based on the intersection genes. Then, key genes were selected by MCODE APP in Cytoscape software.

### Function Enrichment Analysis and Correlation Analysis Between HOXA10 and the Key Genes

To identify the significant biological functions and pathways in which the key genes were involved, Gene Ontology (GO) functional annotation and Kyoto Encyclopedia of Genes and Genomes (KEGG) analysis with the “clusterProfiler” R package were applied. p-value < 0.05 was chosen as the criteria. The correlation between HOXA10 and the key genes were analyzed.

### Validation of mRNA Expression of Six Key Genes in Both HOXA10-Overexpresed ESC and HOXA10-RNAi ESC

We detected the mRNA expression levels of these six genes in HOXA10-overexpressing ESC and HOXA10-knockout ESC by quantitative PCR (qPCR). The total RNA of ectopic and eutopic tissue was extracted according to the RNeasy Plus Mini Kit protocol (Qiagen, Hilden, Germary). According to the Iscript cDNA Synthesis Kit protocol (Bio-Rad, Hercules, CA, USA), cDNA sample were synthesized. qPCR reactions were carried out by the iTaq Univer SYBR Green Supermix Kit protocol (Bio-Rad, Hercules, CA, USA) by repeating each reaction at least three times. The information about the primers is provided in **Supplementary Table S4**.

### Detection of Methylation Rate of HOXA10 CpG Site

According to the reported research (19), we selected three adjacent CpG sites in the third islands, which have only been methylation modified in endometriosis patients. Genomic DNA of both OC and EU tissues were extracted with the QIAamp DNA Mini Kit according to the manufacturer’s instructions (Qiagen, Hilden, Germany). Then, each genomic DNA was converted into bis-DNA with the EZ DNA Methylation-Lightning Kit in accordance with the manufacturer’s protocol (Zymo Research, Irvine, CA, USA). The specific methylation and non-methylation probes for CpGs of interest were designed, and the methylation rates of these CpGs sites were detected by droplet digital PCR (ddPCR). Primers and probes were designed on the basis of the above CpG sites (13). Digital droplet PCR was conducted, and the methylation rate (MR) of each CpGs was calculated. MR (%) = CMS/(CMS + CNMS) × 100% (CMS and CNMS represent the copies for the methylation and non-methylation sites, respectively). The total RNA were extracted, and cDNA was synthesized. qPCR reactions were carried out by the iTaq Univer SYBR Green Supermix Kit protocol (Bio-Rad, Hercules, CA, USA) by repeating each reaction at least three times. To investigate the correlation between the MR of HOXA10 and HOXA10 mRNA expression (**Supplementary Table S5**), the correlation matrix was generated. The primers and probes were listed below:

HOXA10 primer: forward: 5′- TCCGAGAGCAGCAAAGCCT-3′, reverse: 5′-TCCGAGAGCAGCAAAGCCT-3′.

HOXA10 methylation primer: forward: 5′-ATGTTAGGTAATTTTAAAGGTGAA-3′, reverse: 5′-CTTCTCCAACTCCAATATCTAAT-3′, HOXA10 methylation probes: M: 5′-FAM/TGGTCGGAAGAAGCGTTGTTTTTATAC/BHQ1-3′, NM: 5′-HEX/TGGTTGGAAGAAGTGTTGTTTTTATAT/BHQ1-3′. (M, methylation; NM, non-methylation).

### Data Analysis

The statistical analyses performed in this study were conducted in the R environment (version 4.0.3) and GraphPad Prism (version 8.0). The comparison of FPKM of HOXA10 in ESC and OESC was analyzed with t-test. The comparison of mRNA expressions and MRs of HOXA10 in tissue samples was analyzed by GraphPad Prism with non-parametric t-test. The comparison of key genes mRNA expressions was analyzed by t-test. p<0.05 was considered a significant difference. The correlation of Dct and MRs of HOXA10 in tissue samples was analyzed by “corrplor” R package. All R scripts were provided in **Supplementary Data 2**.

## Results

### HOXA10 mRNA Expression in ESC and OESC

**Figure 1:**
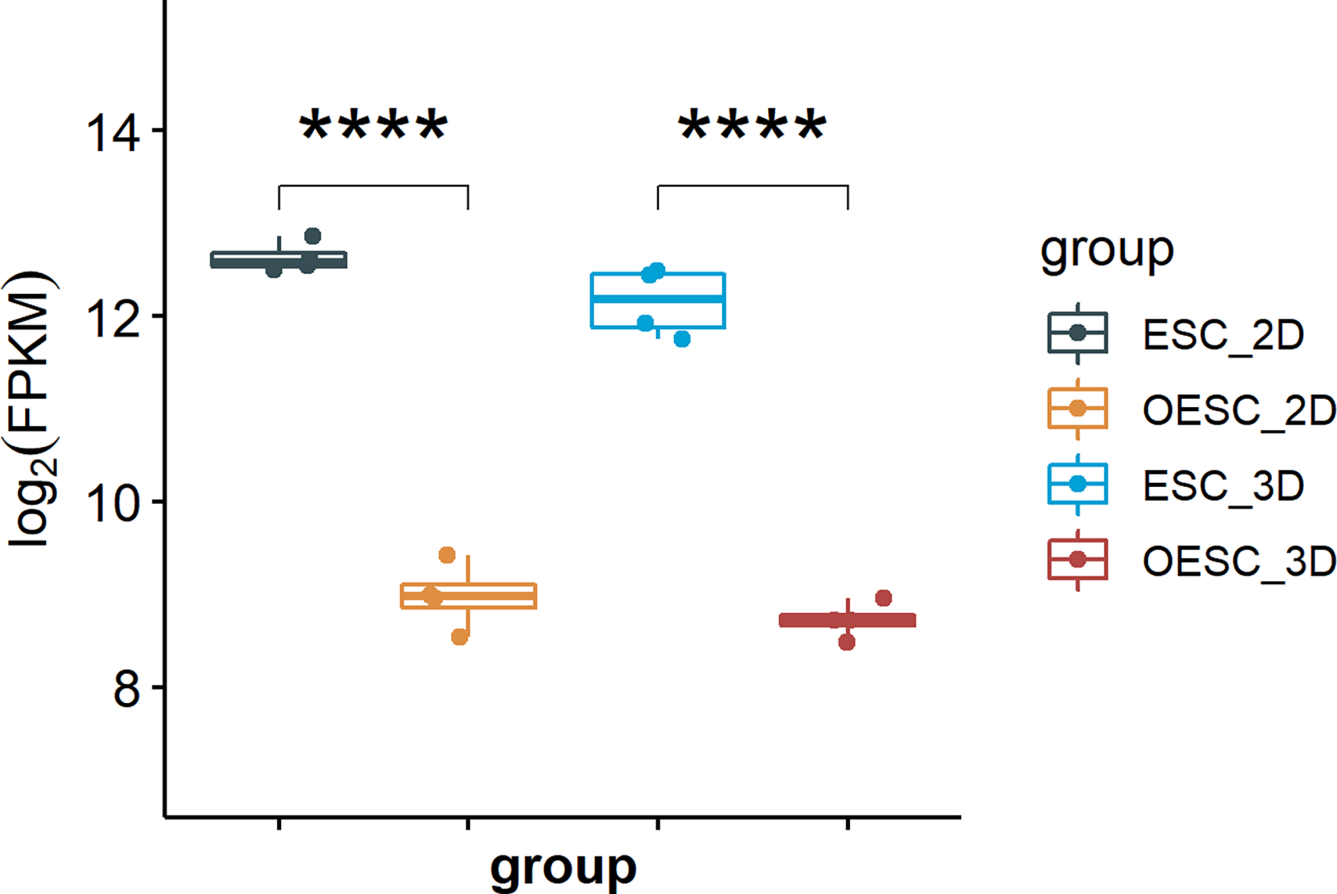
The mRNA expression of HOXA10 in ESC and OESC (ESC_2D, ESC cultured in 2D system; OESC_2D, OESC cultured in 2D system; ESC_3D, ESC cultured in 3D system; OESC_3D, OESC cultured in 3D system. ****p<0.0001).

To compare the expression of HOXA10 in ESC and OESC, we analyzed the download data (GSE136412). The mRNA expression of HOXA10 in ESC was significantly higher than that in OESC no matter in 2D or 3D culture system (p<0.0001 and p<0.0001, respectively) (**Figure 1**).

### The Effects of HOXA10 Over-Expression or Silence in Cultured ESC

**Figure 2:**
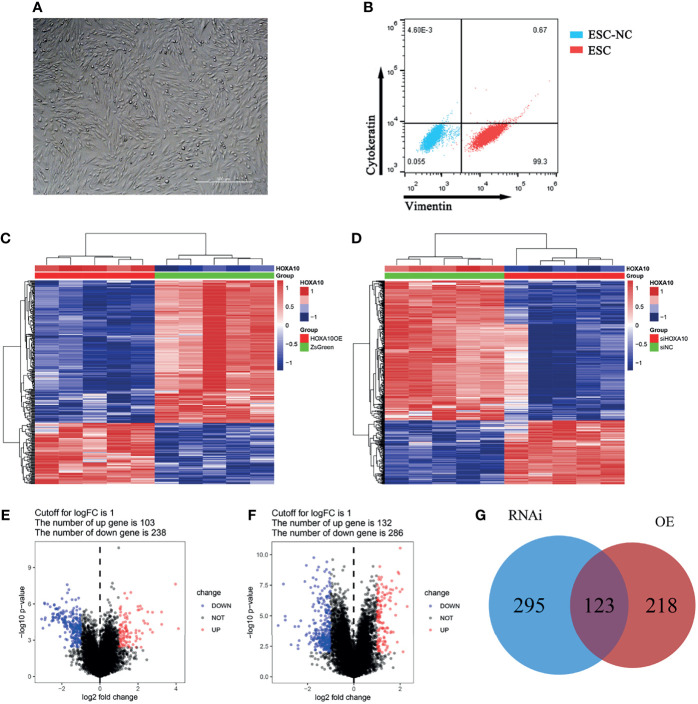
DEGs screening between HOXA10 over-expression and HOXA10 silence ESC. **(A)** ESC culturation. **(B)** ESC identification. **(C)** Heatmap of DEGs between HOXA10OE group and ZsGreen group. **(D)** Heatmap of DEGs between siHOXA10 group and siNC group. **(E)** Volcano plot of DEGs between HOXA10OE group and ZsGreen group. **(F)** DEGs between siHOXA10 group and siNC group. **(G)** The union of two sets of DEGs (HOXA10OE, HOXA10 over-expression ESC; ZsGreen, control for over-expression group; siHOXA10, HOXA10 silence ESC; siNC, control for silence group).

The cultured cells were verified as ESC by flow cytometry, which positively expressed vimentin and negatively expressed cytokeratin (**Figures 2A, B**). mRNA sequencing was conducted in HOXA10-OE ESC (**Figure 2C**) and HOXA10 RNAi ESC (**Figure 2D**). There were 341 DEGs including 103 upregulated genes and 238 downregulated genes in HOXA10 over-expression group (**Figure 2E**). In HOXA10 RNAi group, there were 418 DEGs including 132 upregulated genes and 286 downregulated genes (**Figure 2F**). A union of 636 DEGs that associated with HOXA10 expression were selected after removing the repeated genes (**Figure 2G**).

### Weighted Gene Co-Expression Network Analysis

**Figure 3:**
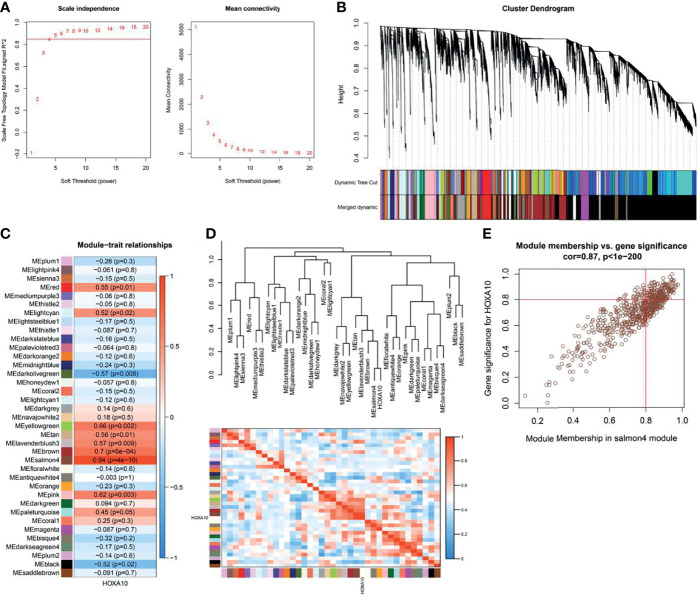
Weighted gene co-expression network analysis. **(A)** Selection of the optimal soft-thresholding power for the scale-free network. **(B)** Module clustering dendrograms. **(C)** Correlation analysis of the module most associated with HOXA10 (Pearson’s correlation coefficient and the corresponding p-value are shown in the horizonal bars). **(D)** Cluster plot of the relationship between HOXA10 and the 37 modules. **(E)** Scatter plot of the genes in salmon4 module showing the relationship between GS and MM (GS, gene significance; MM, module membership).

For screening the HOXA10-associated genes, WGCNA was used to analyze the expression values of 19,225 genes in 20 samples of 4 groups. The soft-thresholding power was 5, which was determined based on a scale-free R^2^ (R^2^ = 0.85) (**Figure 3A**). Therefore, we identified 37 modules when the Diss Thres was set as 0.2 after merging dynamic modules, as shown in the clustering dendrograms (**Figure 3B**). Then, to identify the most HOXA10-related module, we calculated the correlation coefficients between modules and HOXA10 expression. As shown in **Figure 3C**, the salmon4 module exhibited the strongest correlation, with the Pearson correlation of 0.94 and the p-value of 4E−10. **Figure 3D** also indicated that the salmon4 module was most correlated to the HOXA10. Key genes are indicated in the upper-right corner with the threshold of gene significance (GS) > 0.8 and module membership (MM) > 0.8. Finally, for the subsequent analysis, we set the thresholds of GS > 0.8 and MM > 0.8, which allowed us to screen out the final 180 HOXA10-associated genes (**Figure 3E**).

### Screening and Pathway Enrich of Key Gene

**Figure 4:**
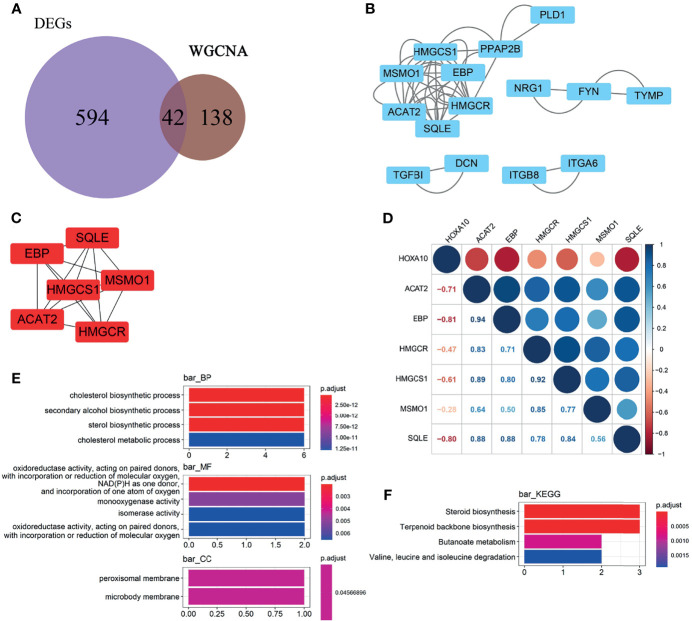
Selection and enrichment analysis of the key genes. **(A)** Venn diagrams showing the candidate genes between the 636 DEGs and 180 HOXA10-associated genes. **(B)** PPI network of the candidate genes. **(C)** Key genes selected by MCODE APP. **(D)** Correlation between the HOXA10 and the six selected key genes. **(E)** GO enrichment analysis of the key genes. **(F)** KEGG enrichment analysis of the key genes (BP, biological process; CC, cellular component; MF, molecular function).

We selected the 42 intersection genes between the 636 DEGs and 180 HOXA10-associated genes (**Figure 4A**). To investigate the key genes that were related to HOXA10, PPIs were constructed in the database (https://www.string-db.org) and visualized by Cytoscape software (version 3.2.1) based on the 42-candidate gene (**Figure 4B**). HMGCR, MSMO1, ACAT2, HMGCS1, EBP, and SQLE were selected by MCODE APP as key genes (**Figure 4C**). The correlation analysis indicated that all these key genes showed significantly negative relationship with HOXA10 (**Figure 4D**). As shown in **Figure 4E**, the six key genes were markedly enriched in the biological process (BP) of cholesterol synthesis pathway by GO analysis; the main related molecular function (MF) terms was oxidoreductase activity, and the main cellular component (CC) was peroxisomal membrane. KEGG analysis demonstrated that the most significantly enriched pathway was steroid biosynthesis and terpenoid backbone biosynthesis (**Figure 4F**).

### The Regulation of HOXA10 on Key Genes

**Figure 5:**
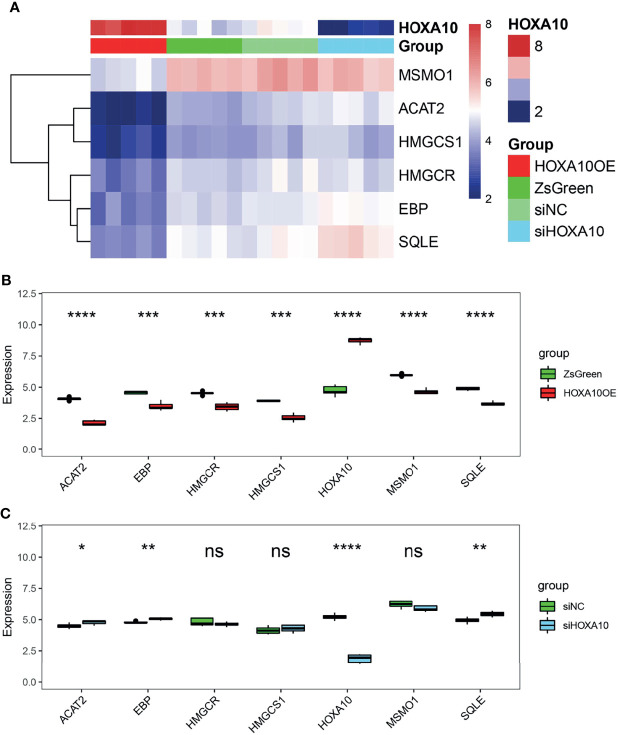
The regulatory effect of HOXA10 on key genes. **(A)** Heatmap of key genes in HOXA10OE, ZsGreen, siHOXA10, and siNC groups. **(B)** The comparison of key genes mRNA expression in HOXA10OE and ZsGreen groups. **(C)** The comparison of key genes mRNA expression in siHOXA10 and siNC groups (HOXA10OE, HOXA10 over expression ESC; ZsGreen, control for over-expression group; siHOXA10, HOXA10 silence ESC; siNC, control for silence group. *p<0.05, **p<0.01, ***p<0.001, ****p<0.0001; ns, no significant difference).

To figure out the details of the HOXA10 regulation effect on key genes, we analyzed the gene expression [log2(fpkm+1)] of both HOXA10 and key genes in four groups (**Figure 5A**). HOXA10 over-expression downregulated all the key genes (**Figure 5B**), and interfering with HOXA10 expression by siRNA up-regulated the expression of ACAT2, EBP, and SQLE (**Figure 5C**).

### Validation of mRNA Expression of Six Key Genes

**Figure 6:**
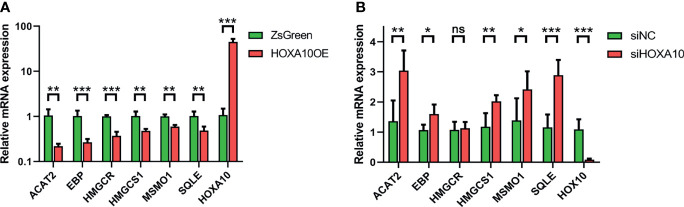
Validation of mRNA expression of six key genes by qPCR. **(A)** The relative mRNA expressions of the six key genes in HOXA10OE and ZsGreen groups. **(B)** The relative mRNA expressions of the six key genes in siHOXA10 and siNC groups (HOXA10OE, HOXA10 over expression ESC; ZsGreen, control for HOXA10OE; siHOXA10, HOXA10 silence ESC; siNC, control for silence group; *p<0.05, **p<0.01, ***p<0.001. ns, no significant difference).

We verified the mRNA expression levels of key genes in the above four groups of ESC by qPCR. As shown in **Figure 6**, all six genes were down-regulated in HOXA10-overexpresed ESC (**Figure 6A**). In addition, five of the six key genes were upregulated in HOXA10-RNAi ESC except for HMGCR (**Figure 6B**). The qPCR results confirmed the results of mRNA sequencing.

### Comparation of the Methylation Rate of HOXA10 in EU and OC Tissues

**Figure 7:**
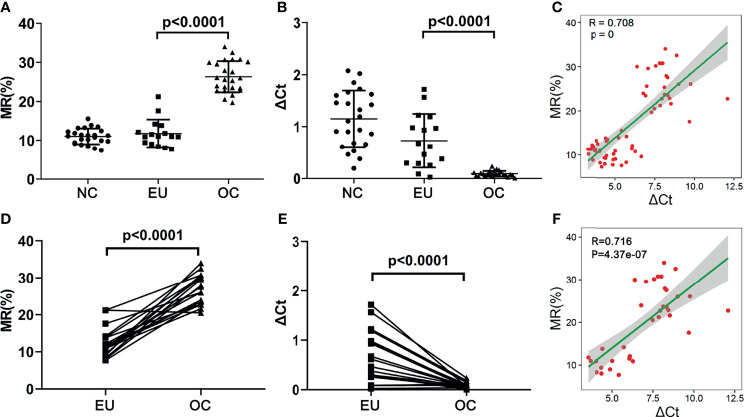
The MRs and mRNA expression of HOXA10 in NC, EU and OC. **(A)** The MRs of representative CpGs detected by ddPCR. **(B)** The mRNA expression of HOXA10 in EU and OC. **(C)** The correlation between MRs and HOXA10 mRNA expression. **(D)** The comparisons of MR in EU and OC from the same patients. **(E)** The comparisons of HOXA10 mRNA expression in EU and OC from the same patients. **(F)** The correlation between MRs and HOXA10 mRNA expressions in EU and OC from the same patients (MR, methylation rate; EU, eutopic endometrial tissues; OC, ovarian cyst; NC, normal control).

We collected surgical endometrial tissue samples from patients with non-endometriosis-related disease [negative control (NC); n = 24], surgical eutopic endometrial tissue samples from patients with OEMs (EU; n = 16), and ovarian cyst (the chocolate-colored part of the tissue) (OC; n = 24). We further verified the MR of the selected CpGs in both OC and EU tissue. As shown in **Figure 7**, the MR of the representative sites in OC was remarkably higher than that in EU (p<0.0001) (**Figure 7A**), while the expression of HOXA10 in OC was notably lower than that in EU (p<0.0001) (**Figure 7B**). The methylation rates of the representative CpGs were positively correlated with ΔCt values HOXA10 mRNA expressions (R=0.708) (**Figure 7C**). Results of EU and OC from the same patient showed that the MR of the representative sites in OC was also remarkably higher than that in EU (p<0.0001) (**Figure 7D**), the expression of HOXA10 in OC was also notably lower than that in EU (p<0.0001) (**Figure 7E**). The methylation rates of the representative CpGs were positively correlated with ΔCt values HOXA10 mRNA expressions (R=0.716) (**Figure 7F**).

## Discussion

HOXA10 is highly expressed in endometrial stromal cells and plays a key role in the proliferation and differentiation of the endometrium (23). However, the expression of HOXA10 is significantly diminished in ectopic lesions of OEMs (24). What is more, patients with endometriosis have a significantly increased risk of diseases, which is probably due to the lipids (total cholesterol, LDL-C) (25). It is reported that the expression of HOXA10 in patients with polycystic ovary is positively correlated with the concentrations of blood HDL and is negatively correlated with the concentrations of blood TG, TC, and LDL (11). Therefore, HOXA10 may play a regulatory role in the steroid hormone–cholesterol synthesis pathway of endometrial stromal cells. The expression of HOXA10 in ESC is regulated by the level of steroid hormones (10, 26), but the regulation mechanism is still unclear. In patients with OEMs, the expression of HOXA10 in ectopic lesions is diminished or significantly downregulated (24, 27). In patients with OEMs, it is accompanied by abnormal cholesterol metabolism. As cholesterol is the parent molecule of sterol hormones, HOXA10 may play a regulatory role in the steroid hormone–cholesterol synthesis pathway of endometrial stromal cells. The expression of HOXA10 is diminished in ectopic endometrium. Abnormal cholesterol metabolism in patients with ovarian endometriosis may be related to the abnormal low expression of HOXA10.

In our study, we found that HOXA10 mRNA expression in ESC was significantly lower than that in OESC. Six genes (HMGCOR, MSMO1, ACAT2, HMGCS1, EBP, and SQLE) were screened and markedly enriched in cholesterol and secondary alcohol synthesis pathway (**Figures 4C–F**). HMG-CoA reductase (HMGCR), as the rate-limiting enzyme for cholesterol synthesis, catalyzes the formation of mevalonate, which is a key intermediate for sterol synthesis (28, 29). The 3-hydroxy-3-methylglutaryl-CoA synthase 1 (HMGCS1) works as a potential gatekeeper in the mevalonate pathway (30). Squalene epoxidase (SQLE) is also one of the rate-limiting enzymes in the cholesterol biosynthesis (31). MSMO1 is the oxidase-encoding gene, catalyzing demethylation of C4-methylsterols, which was named as meiosis activating and stimulated cell over proliferation (32). The protein encoded by gene EBP is an ER-localized protein, which is also a 3-beta hydroxysteroid-delta (8) and delta (7)-isomerase and essential for sterol biosynthesis in eukaryotic cells (33). ACAT1 encodes an enzyme that synthesizes cholesterol and long-chain fatty acids into cholesterol esters, which is transported to the circulatory system through cholesterol ester transfer protein (34). Therefore, we proposed that HOXA10 may regulate cholesterol synthesis in endometrial stromal cells.

All key genes were downregulated by HOXA10 over-expression, and at least three key genes (ACAT2, EBP, and SQLE) were upregulated by HOXA10 silence. Compared with the cholesterol synthesis promoted by HOXA10 downregulation, the upregulation of HOXA10 inhibited the synthesis of cholesterol more significantly. Hence, we proposed that HOXA10 acts as an inhibitor of cholesterol synthesis, and the low expression of HOXA10 in ectopic lesions loses its regulatory effect on cholesterol synthesis. Additionally, we also found that MRs of the representative CpGs was significantly related to HOXA10 mRNA expression in endometrial tissue. Therefore, HOXA10 DNA methylation modification be involved in regulating its mRNA expression. As one of the epigenetic modification, DNA methylation modification is the early event of diseases (35), indicating that HOXA10 methylation may be the early indicator of endometriosis. It should be noted that chocolate cysts contain very few endometriotic tissues (36), so further purification of the ovarian ectopic endometrial tissue is required to illustrate the relationship between HOXA10 mRNA and methylation rates.

It was reported that steroid hormone regulates the expression of HOXA10 *in vivo* and *in vitro* (37). After binding to estradiol, the estrogen receptor binds to the estrogen response elements (EREs) of HOXA10 to upregulate its expression (10). In the primary endometrial cell from healthy volunteers, the expression of HOXA10 is significantly upregulated after being treated with 17β-estradiol (9). Considering that cholesterol is the parent molecule of estrogen and HOXA10 regulated the synthesis of cholesterol in ESC, we speculated that HOXA10 plays an important negative feedback regulation role in the pathway of cholesterol and estrogen metabolism. After estrogen upregulated the expression of HOXA10, the synthesis of cholesterol was inhibited by downregulating the six key genes of cholesterol synthesis, thereby reducing the synthesis of estrogen parent molecules. Therefore, for patients with OEMs, the abnormal expression of HOXA10 in OESC reduced the inhibitory effect on the cholesterol synthesis pathway, leading to an increased risk of sterol metabolism diseases. The cholesterol abnormal metabolism has been observed to be correlated with the endometriosis (16). Mu et al. reported a strong association between confirmed EMs and hypercholesterolemia and hypertension in a large prospective cohort study (17). We also proposed that patients treated with GnRH antagonist may also need to consider the use of drugs that inhibit cholesterol accumulation. HOXA10 and its methylation modification sites may become one of the potential therapeutic targets for OEMs.

However, our study was not without its shortcomings. We have not verified the regulatory effect of HOXA10 on the six key genes in ectopic endometrial stromal cells because we have not obtained target cells that can be passaged stably. Additionally, whether HOXA10 directly or indirectly affects these six key genes needs to be verified by Hi-C or Chip-seq. The correlation between methylation and HOXA10 expression needs to be verified by methylation editing in our future study.

In summary, we found that HOXA10 regulates the cholesterol synthesis in ESC through the six key genes. We speculated that the relatively high expression of HOXA10 in normal endometrium inhibits the excessive synthesis of cholesterol. On the contrary, the relatively low expression of HOXA10 in ectopic lesions may lead to the loss of proper inhibition of cholesterol synthesis in the patients with OEMS. In addition, the expression of HOXA10 may be regulated by the methylation modification on HOXA10 CpGs.

## Data Availability Statement

The datasets presented in this study can be found in online repositories. The names of the repository/repositories and accession number(s) can be found in the article/**Supplementary Material**.

## Ethics Statement

The studies involving human participants were reviewed and approved by the Ethics Committee of Guangzhou Women and Children’s Medical Center, Guangzhou, China (Permit Approval No. 2017102709). The patients/participants provided their written informed consent to participate in this study.

## Author Contributions

MY designed the research and revised the language of the paper. JT, CG, PD, and NL collected the research data. YH sorted the patients’ information. QL analyzed the data and wrote the draft. All authors contributed to the article and approved the submitted version.

## Conflict of Interest

The authors declare that the research was conducted in the absence of any commercial or financial relationships that could be construed as a potential conflict of interest.

## Publisher’s Note

All claims expressed in this article are solely those of the authors and do not necessarily represent those of their affiliated organizations, or those of the publisher, the editors and the reviewers. Any product that may be evaluated in this article, or claim that may be made by its manufacturer, is not guaranteed or endorsed by the publisher.

## Supplementary Material

Supplementary Table 1 Clinical characteristics of the study participants.

Supplementary Table 2 HOXA10 mRNA expression level in ESC and OESC.

Supplementary Table 3 The FPKM of HOXA10 over-expression and HOXA10 silence in ESC.

Supplementary Table 4 The qPCR primers for mRNA expression of the six genes.

Supplementary Table 5 The data of MR and mRNA expression of HOXA10 in tissue samples.

Supplementary Data Sheet 1 The flow cytometry data and gating strategies.

Supplementary Data Sheet 2 The R script.
