# Supplementary figures and images for: HOXA10 Regulates the Synthesis of Cholesterol in Endometrial Stromal Cells
Source: Front Endocrinol (Lausanne). 2022 Apr 25;13:852671. doi: 10.3389/fendo.2022.852671 (PMC9084188; doi:10.3389/fendo.2022.852671)

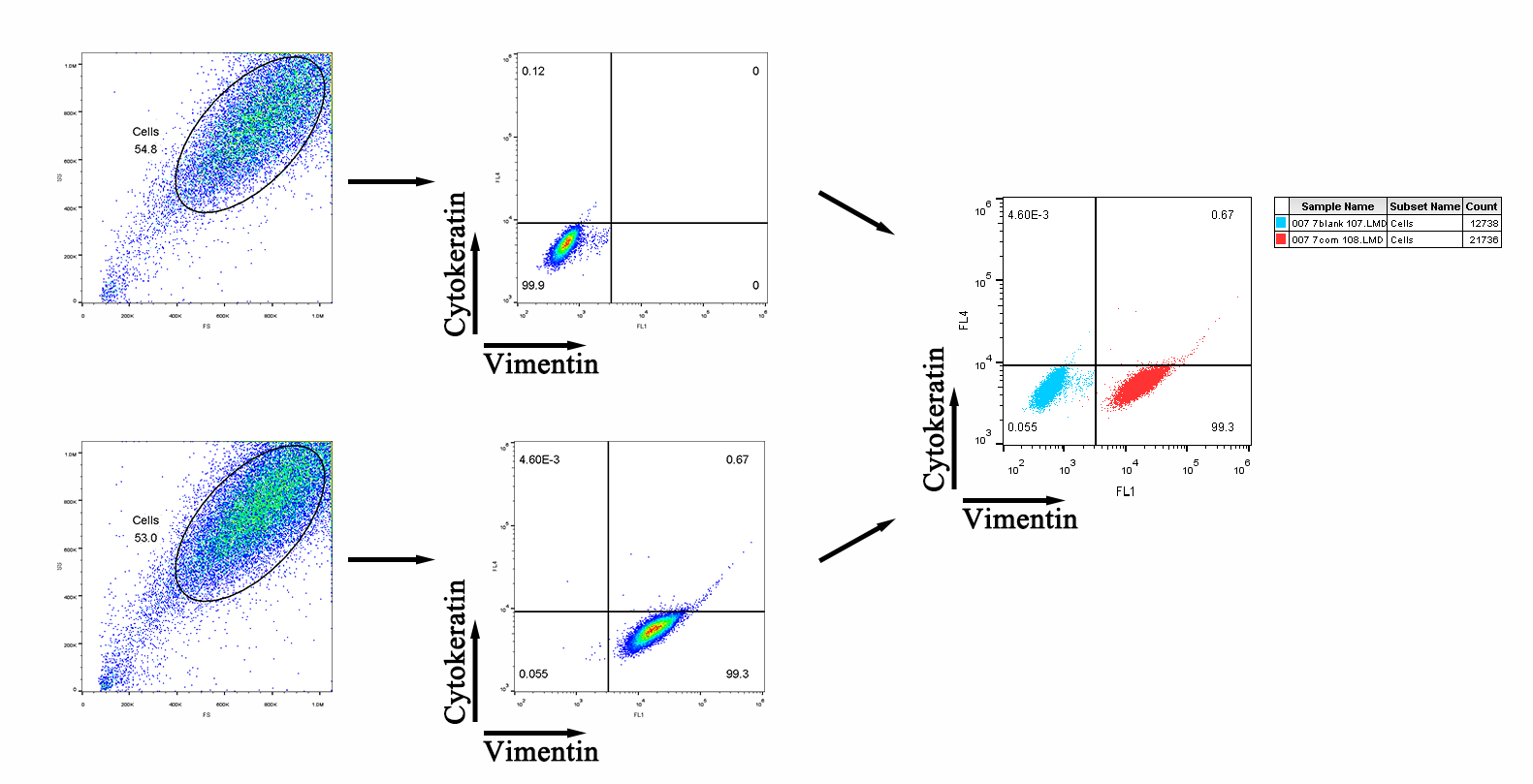

Supplement: Supplementary Data Sheet 1 — The flow cytometry data and gating strategies. [file DataSheet_1.zip › ESC_flow gating strategies.tif]
