# Supplementary material for: HOXA10 Regulates the Synthesis of Cholesterol in Endometrial Stromal Cells
Source: Front Endocrinol (Lausanne). 2022 Apr 25;13:852671. doi: 10.3389/fendo.2022.852671 (PMC9084188; doi:10.3389/fendo.2022.852671)
Supplement: Supplementary Data Sheet 2 — The R script. [file DataSheet_2.doc]

###HOXA10 over expression##################################################################

rm(list = ls())

options(stringsAsFactors = F)

getwd()

setwd("H:/HOXA10/HOXA10 数据/")

a<-read.csv("hoxa10 EGFP HOXA10.csv")

head(a[1:4,1:4])

b<-colnames(a)

rownames(a)<-a[,1]

a1<-a[,-1]

head(a1[1:4,1:4])

group<-read.csv("group.csv")

#

qx1 <- as.numeric(quantile(a1, c(0., 0.25, 0.5, 0.75, 0.99, 1.0), na.rm=T))

View(qx1)

LogC <- (qx1[5] > 100) ||

(qx1[6]-qx1[1] > 50 && qx1[2] > 0) ||

(qx1[2] > 0 && qx1[2] < 1 && qx1[4] > 1 && qx1[4] < 2)

LogC#

#

a2<-log2(a1+1)

qx1 <- as.numeric(quantile(a2, c(0., 0.25, 0.5, 0.75, 0.99, 1.0), na.rm=T))

View(qx1)

LogC <- (qx1[5] > 100) ||

(qx1[6]-qx1[1] > 50 && qx1[2] > 0) ||

(qx1[2] > 0 && qx1[2] < 1 && qx1[4] > 1 && qx1[4] < 2)

LogC#

#

library("FactoMineR")

library("factoextra")

library("ggplot2")

df.pca <- PCA(t(a2))

df.pca <- PCA(t(a2), graph = FALSE)

fviz_pca_ind(df.pca,

geom.ind = "point",

col.ind = group$group,

addEllipses = TRUE,

legend.title = "Groups")

group_list<-group$group

save(a2,group_list,file='PCAOA.Rdata')

#

load('PCAOA.Rdata')

design <- model.matrix(~0+factor(group_list))

a1<-a2

colnames(design)=levels(factor(group_list))

rownames(design)=colnames(a1)

library(limma)

contrast.matrix<-makeContrasts(contrasts=c('HOXA10-EGFP'),levels = design)

fit <- lmFit(a1,design)

fit1 <- contrasts.fit(fit, contrast.matrix)

fit2 <- eBayes(fit1)

class(fit2)

tT <- topTable(fit2, adjust="fdr", number=nrow(fit2))

tT2 <- topTable(fit2, number=nrow(fit2))

table(tT==tT2)

tT <- subset(tT, select=c('adj.P.Val',"P.Value","logFC"))

save(tT,file='HOXtT.Rdata')

#

load(file = "HOXtT.Rdata")

head(tT[1:4,1:3])

logFC_cutoff <-1

mean(tT$adj.P.Val)

fivenum((tT$adj.P.Val))

mean(abs(tT$logFC))

fivenum(( tT$logFC))

TTV<-tT

TTV$change=ifelse(TTV$adj.P.Val<0.05&abs(TTV$logFC)>logFC_cutoff,ifelse(TTV$logFC > logFC_cutoff ,'UP','DOWN'),'NOT')

table(TTV$change)

this_tile <- paste0('Cutoff for logFC is ',round(logFC_cutoff,3),

'\nThe number of up gene is ',nrow(TTV[TTV$change =='UP',]) ,

'\nThe number of down gene is ',nrow(TTV[TTV$change =='DOWN',]))

write.csv(TTV,"TTV.csv")

TTV<-read.csv("TTV.csv",header=T)

names(TTV)[1]<-"gene_name"

head(TTV[1:4,1:5])

#

rownames(TTV)<-TTV[,1]

TTV<-TTV[,-1]

head(TTV[1:4,1:4])

library(ggplot2)

g <- ggplot(data=TTV, aes(x=logFC, y=-log10(P.Value), color=change)) +geom_point(alpha=0.4, size=1.25) +

theme_set(theme_set(theme_bw(base_size=20)))+xlab("log2 fold change") + ylab("-log10 p-value")+

ggtitle( this_tile ) + theme(plot.title = element_text(size=15,hjust = 0.5))+

scale_colour_manual(values = c('blue','black','red'))+

annotate('text',x=TTV$logFC[TTV$logFC>10],y=-log10(TTV$P.Value[TTV$logFC>10]),

label=rownames(TTV)[TTV$logFC>10])

g1<-g+theme_bw()+theme(panel.grid.major=element_blank(),panel.grid.minor = element_blank())+

geom_vline(xintercept=0, linetype="dashed", color = "black",size=1)

ggsave(g1,filename = 'volcanoHOXOE.png',width=4.5,height = 4)

TTVOE<-TTV[which(TTV$change!="NOT"),]

save(TTVOE,file='volcanoHOXOE.Rdata')

#

load('PCAOA.Rdata')

load('volcanoHOXOE.Rdata')

load('HOXtT.Rdata')

table(group_list)

group_list[which(group_list=="EGFP")]<-"ZsGreen"

group_list[which(group_list=="HOXA10")]<-"HOXA10OE"

table(group_list)

choose_gene<-rownames(TTVOE)

data_matrix0 <-a2[choose_gene,]

data_matrix=t(scale(t(data_matrix0)))

fivenum(data_matrix)

data_matrix[data_matrix>1.2]=1.2

data_matrix[data_matrix< -1.2] = -1.2

library(pheatmap)

library(RColorBrewer)

bk = c(seq(-1.2,1.2, length=100)) #

annotation_col = data.frame(Group = group_list,

HOXA10 = data_matrix[which(rownames(data_matrix)=="HOXA10"),])

ann_colors = list(Group = c(HOXA10OE= "red", ZsGreen = "green"),

HOXA10 = colorRampPalette(c("navy", "white", "firebrick3"))(100))

pheatmap(data_matrix,

breaks=bk,

legend_breaks=c(-1,-0.5,0,0.5,1),

show_rownames = F,

show_colnames = F,

cluster_col = T,

cluster_row = T,

border = NA,

cellheight = 1,

cellwidth = 40,

fontsize = 10,

fontsize_row = 10,

fontsize_col = 10,

#display_numbers = p21,

annotation_col = annotation_col,

annotation_colors = ann_colors,

color = colorRampPalette(c("navy", "white", "firebrick3"))(100),

filename = 'pheatmaphoxaOE.pdf',width = 10,height = 8 )

###HOXA10 siRNA#########################################################################

rm(list = ls())

options(stringsAsFactors = F)

getwd()

setwd("H:/HOXA10/HOXA10 RNAI 数据/")

a<-read.csv("HOXA10 RNAI 02.csv")

head(a[1:4,1:4])

b<-colnames(a)

rownames(a)<-a[,1]

a1<-a[,-1]

head(a1[1:4,1:4])

group<-read.csv("group.csv")

#

qx1 <- as.numeric(quantile(a1, c(0., 0.25, 0.5, 0.75, 0.99, 1.0), na.rm=T))

View(qx1)

LogC <- (qx1[5] > 100) ||

(qx1[6]-qx1[1] > 50 && qx1[2] > 0) ||

(qx1[2] > 0 && qx1[2] < 1 && qx1[4] > 1 && qx1[4] < 2)

LogC

#

a2<-log2(a1+1)

qx1 <- as.numeric(quantile(a2, c(0., 0.25, 0.5, 0.75, 0.99, 1.0), na.rm=T))

View(qx1)

LogC <- (qx1[5] > 100) ||

(qx1[6]-qx1[1] > 50 && qx1[2] > 0) ||

(qx1[2] > 0 && qx1[2] < 1 && qx1[4] > 1 && qx1[4] < 2)

LogC#

#

library("FactoMineR")

library("factoextra")

library("ggplot2")

df.pca <- PCA(t(a2))

df.pca <- PCA(t(a2), graph = FALSE)

fviz_pca_ind(df.pca,

geom.ind = "point",

col.ind = group$group,

addEllipses = TRUE,

legend.title = "Groups")

group_list<-group$group

save(a2,group_list,file='PCAOA.Rdata')

#

load('PCAOA.Rdata')

design <- model.matrix(~0+factor(group_list))

a1<-a2

colnames(design)=levels(factor(group_list))

rownames(design)=colnames(a1)

library(limma)

contrast.matrix<-makeContrasts(contrasts=c('siHOX2-RNAiNC'),levels = design)

fit <- lmFit(a1,design)

fit1 <- contrasts.fit(fit, contrast.matrix)

fit2 <- eBayes(fit1)

class(fit2)

tT <- topTable(fit2, adjust="fdr", number=nrow(fit2))

tT2 <- topTable(fit2, number=nrow(fit2))

table(tT==tT2)

tT <- subset(tT, select=c('adj.P.Val',"P.Value","logFC"))

save(tT,file='HOXtT.Rdata')

#

load(file = "HOXtT.Rdata")

logFC_cutoff <-1

mean(tT$adj.P.Val)

fivenum((tT$adj.P.Val))

mean(abs(tT$logFC))

fivenum(( tT$logFC))

TTV<-tT

TTV$change=ifelse(TTV$adj.P.Val<0.05&abs(TTV$logFC)>logFC_cutoff,ifelse(TTV$logFC > logFC_cutoff ,'UP','DOWN'),'NOT')

table(TTV$change)

this_tile <- paste0('Cutoff for logFC is ',round(logFC_cutoff,3),

'\nThe number of up gene is ',nrow(TTV[TTV$change =='UP',]) ,

'\nThe number of down gene is ',nrow(TTV[TTV$change =='DOWN',]))

write.csv(TTV,"TTV.csv")

TTV<-read.csv("TTV.csv",header=T)

names(TTV)[1]<-"gene_name"

head(TTV[1:4,1:4])

#

rownames(TTV)<-TTV[,1]

TTV<-TTV[,-1]

head(TTV[1:4,1:4])

library(ggplot2)

g <- ggplot(data=TTV, aes(x=logFC, y=-log10(P.Value), color=change)) +geom_point(alpha=0.4, size=1.25) +

theme_set(theme_set(theme_bw(base_size=20)))+xlab("log2 fold change") + ylab("-log10 p-value") +

ggtitle( this_tile ) + theme(plot.title = element_text(size=15,hjust = 0.5))+

scale_colour_manual(values = c('blue','black','red'))+

annotate('text',x=TTV$logFC[TTV$logFC>5],y=-log10(TTV$P.Value[TTV$logFC>5]),label=rownames(TTV)[TTV$logFC>5])

g1<-g+theme_bw()+ theme(panel.grid.major = element_blank(),panel.grid.minor = element_blank())+

geom_vline(xintercept=0, linetype="dashed", color = "black",size=1)

ggsave(g1,filename = 'volcanoHOXRNAI.pdf',width=4.5,height = 4)

TTVRNAI<-TTV[which(TTV$change!="NOT"),]

save(TTVRNAI,file='volcanoHOXRNAI.Rdata')

#

load('PCAOA.Rdata')

load('volcanoHOXRNAI.Rdata')

load('HOXtT.Rdata')

table(group_list)

group_list[which(group_list=="RNAiNC")]<-"siNC"

group_list[which(group_list=="siHOX2")]<-"siHOXA10"

table(group_list)

choose_gene<-rownames(TTVRNAI)

data_matrix0 <-a2[choose_gene,]

data_matrix=t(scale(t(data_matrix0)))

fivenum(data_matrix)

data_matrix[data_matrix>1.2]=1.2

data_matrix[data_matrix< -1.2] = -1.2

library(pheatmap)

library(RColorBrewer)

bk = c(seq(-1.2,1.2, length=100))

annotation_col = data.frame(Group = group_list,

HOXA10 = data_matrix[which(rownames(data_matrix)=="HOXA10"),])

ann_colors = list(Group = c(siHOXA10= "red", siNC = "green"),

HOXA10 = colorRampPalette(c("navy", "white", "firebrick3"))(100))

pheatmap(data_matrix,

breaks=bk,

legend_breaks=c(-1,-0.5,0,0.5,1),

show_rownames = F,

show_colnames = F,

cluster_col = T,

cluster_row = T,

border = NA,

cellheight = 0.8157,

cellwidth = 40,

fontsize = 10,

fontsize_row = 10,

fontsize_col = 10,

#display_numbers = p21,

annotation_col = annotation_col,

annotation_colors = ann_colors,

color = colorRampPalette(c("navy", "white", "firebrick3"))(100),

filename = 'pheatmapSIRNAhoxa10.pdf',width = 10,height = 8 )

########WGCNA###########################################################

HOX<-read.csv("hoxa10 EGFP HOXA10.csv",header = T)

SI<-read.csv("HOXA10 RNAI 02.csv",header = T)

hoxsi<-merge(HOX,SI,by="GeneID")

head(hoxsi[1:4,1:4])

rownames(hoxsi)<-hoxsi[,1]

hoxsi1<-hoxsi[,-1]

head(hoxsi1[1:4,1:4])

hoxsi2<-as.data.frame(t(hoxsi1))

head(hoxsi2[1:4,1:4])

w<-which(colnames(hoxsi2)=="HOXA10")

phoxa10<-hoxsi2[,c(1,w)]

phoxa10[,1]<-rownames(phoxa10)

names(phoxa10)[1]<-"sample"

save(hoxsi2,phoxa10,file='data1.Rdata')

#

library(WGCNA)

library(data.table)

library(stringr)

library(openxlsx)

allowWGCNAThreads()

ALLOW_WGCNA_THREADS=4

memory.limit(size = 20000)

load("data1.Rdata")

data2<-hoxsi2

exprSize = checkSets(data2,checkStructure = T)

gsg = goodSamplesGenes(data2)

gsg$allOK#若为F，需要进行下一步筛选

if (!gsg$allOK) {

data2 = data2[gsg$goodSamples, gsg$goodGenes]

}

gsg = goodSamplesGenes(data2)

gsg$allOK

#

nGenes = ncol(data2)

nSamples = nrow(data2)

save(data2,file='data2.Rdata')

powers = c(c(1:10), seq(from = 12, to=20, by=2))

sft = pickSoftThreshold(data2, powerVector = powers, verbose = 5)

pdf("001SFT.pdf",width=10,height = 6)

par(mfrow = c(1,2));

cex1 = 0.9;

plot(sft$fitIndices[,1], -sign(sft$fitIndices[,3])*sft$fitIndices[,2],

xlab="Soft Threshold (power)",ylab="Scale Free Topology Model Fit,signed R^2",type="n",

main = paste("Scale independence"));

text(sft$fitIndices[,1], -sign(sft$fitIndices[,3])*sft$fitIndices[,2],

labels=powers,cex=cex1,col="red");

abline(h=0.85,col="red")

plot(sft$fitIndices[,1], sft$fitIndices[,5],

xlab="Soft Threshold (power)",ylab="Mean Connectivity", type="n",

main = paste("Mean connectivity"))

text(sft$fitIndices[,1], sft$fitIndices[,5], labels=powers, cex=cex1,col="red")

dev.off()

#

softPower <- sft$powerEstimate

softPower

adjacency = adjacency(data2, power = softPower);

TOM = TOMsimilarity(adjacency);

dissTOM = 1-TOM

hierTOM = hclust(as.dist(dissTOM),method="average")

#

ADJ1_cor <- abs(WGCNA::cor( data2,use = "p" ))^softPower

k <- softConnectivity(datE=data2,power=softPower)

pdf("002K.pdf",width=10,height = 6)

par(mfrow = c(1,2))

hist(k)

scaleFreePlot(k,main="Check Scale free topology\n")

dev.off()

save(softPower,file='softPower.Rdata')

geneTree = hclust(as.dist(dissTOM), method = "average");

pdf("003Tree.pdf",width=10,height = 6)

plot(geneTree, xlab="", sub="", main = "Gene clustering on TOM-based dissimilarity",

labels = FALSE, hang = 0.04);

minModuleSize = 30;

dynamicMods = cutreeDynamic(dendro = geneTree, distM = dissTOM,

deepSplit = 2, pamRespectsDendro = FALSE,

minClusterSize = minModuleSize);

table(dynamicMods)

#

dynamicColors <- labels2colors(dynamicMods)

table(dynamicColors)

plotDendroAndColors(geneTree, dynamicColors, 'Dynamic Tree Cut',

dendroLabels = FALSE, addGuide = TRUE, hang = 0.03, guideHang = 0.05,

main = 'Gene dendrogram and module colors')

dev.off()

save(dynamicMods,file='dynamicMods.Rdata')

#

MEList = moduleEigengenes(data2, colors = dynamicColors)

MEs = MEList$eigengenes

MEDiss = 1-cor(MEs);

METree = hclust(as.dist(MEDiss), method = "average")

MEDissThres = 0.2

merge_modules = mergeCloseModules(data2, dynamicColors, cutHeight = MEDissThres, verbose = 3)

mergedColors = merge_modules$colors;

mergedMEs = merge_modules$newMEs;

pdf("007mergedTree.pdf",width=10,height = 6)

plotDendroAndColors(geneTree, cbind(dynamicColors, mergedColors),

c("Dynamic Tree Cut", "Merged dynamic"),

dendroLabels = FALSE, hang = 0.03,

addGuide = TRUE, guideHang = 0.05)

dev.off()

save(mergedMEs,file='mergedMEs.Rdata')

save(mergedColors,file='mergedColors.Rdata')

save(MEs,file='MEs.Rdata')

#

library(WGCNA)

library(data.table)

library(stringr)

library(openxlsx)

#

load("data1.Rdata")

load("mergedMEs.Rdata")

load("data2.Rdata")

load("mergedColors.Rdata")

load("softPower.Rdata")

table(mergedColors)

pho101<-read.csv("phoxa10.csv",header = T)

rownames(pho101)<-pho101$sample

pho6<-pho101[,-c(1,2)]

save(pho6,file = 'pho6.Rdata')

colnames(pho6)

nGenes = ncol(data2)

nSamples = nrow(data2)

x<-which(colnames(pho6)=="HOXA10")

pdf("015Intramodular Connectivity and Module Membership02.pdf",width=5,height = 10)

moduleTraitCor_noFP <- cor(mergedMEs, pho6[,x], use = "p");

moduleTraitPvalue_noFP = corPvalueStudent(moduleTraitCor_noFP, nSamples);

textMatrix_noFP <- paste(signif(moduleTraitCor_noFP, 2), " (p=", signif(moduleTraitPvalue_noFP, 1), ")", sep = "");

par(mar = c(6, 10, 3, 2));#下、左、上、右

labeledHeatmap(Matrix = moduleTraitCor_noFP,

xLabels = names(pho6)[x],

xLabelsAngle = 0,

xLabelsAdj = 0.5,

yLabels = names(mergedMEs),

ySymbols = names(mergedMEs),

colorLabels = FALSE,

colors = blueWhiteRed(50),

textMatrix = textMatrix_noFP,

setStdMargins = FALSE,

#cex.text = 0.65,

zlim = c(-1,1),

main = paste("Module-trait relationships"))

dev.off()

cor_ADR <- signif(WGCNA::cor(pho6,mergedMEs,use="p",method="pearson"),5)

p.values <- corPvalueStudent(cor_ADR,nSamples=nrow(pho6))

#

colnames(pho6)

colnames(cor_ADR)

Freq_MS_max_cor <- which.max(abs(cor_ADR[colnames(pho6)[x],-which(colnames(cor_ADR) == "MEgrey")]))

Freq_MS_max_p <- which.min(p.values[colnames(pho6)[x],-which(colnames(p.values) == "MEgrey")])

Freq_MS_max_cor

Freq_MS_max_p

GS1 <- as.numeric(WGCNA::cor(pho6[,colnames(pho6)[x]],data2,use="p",method="pearson"))

GeneSignificance <- abs(GS1)

ModuleSignificance <- tapply(GeneSignificance,mergedColors,mean,na.rm=T)

ModuleSignificance

which.max(ModuleSignificance[names(ModuleSignificance)!="grey"])

maxmodule<-which.max(ModuleSignificance[names(ModuleSignificance)!="grey"])

maxmodule1<-as.data.frame(maxmodule)

scolor<-rownames(maxmodule1)

ADJ1=abs(cor(data2,use="p"))^softPower

Alldegrees1=intramodularConnectivity(ADJ1, moduleColors)

datKME=signedKME(data2, mergedMEs, outputColumnName="MM.")

Hoxa10 = as.data.frame(pho6[x]);

names(Hoxa10 ) = colnames(pho6)[x]

# Add the weight to existing module eigengenes

MET = orderMEs(cbind(mergedMEs, Hoxa10 ))

# Plot the relationships among the eigengenes and the trait

pdf("017Hoxa10 .pdf",width=10,height = 10);#下、左、上、右

par(cex = 0.55)

plotEigengeneNetworks(MET, "", marDendro = c(0,7,1,2), marHeatmap = c(6,7,1,2), cex.lab = 0.8,

xLabelsAngle= 90)

dev.off()

#

corType="pearson"

if (corType=="pearson") {

geneModuleMembership = as.data.frame(cor(data2, mergedMEs, use = "p"))

MMPvalue = as.data.frame(corPvalueStudent(

as.matrix(geneModuleMembership), nSamples))

} else {

geneModuleMembershipA = bicorAndPvalue(data2, mergedMEs, robustY=ifelse(corType=="pearson",T,F))

geneModuleMembership = geneModuleMembershipA$bicor

MMPvalue = geneModuleMembershipA$p

}

if (corType=="pearson") {

geneTraitCor = as.data.frame(cor(data2, pho6, use = "p"))

geneTraitP = as.data.frame(corPvalueStudent(

as.matrix(geneTraitCor), nSamples))

} else {

geneTraitCorA = bicorAndPvalue(data2, pho6, robustY=ifelse(corType=="pearson",T,F))

geneTraitCor = as.data.frame(geneTraitCorA$bicor)

geneTraitP = as.data.frame(geneTraitCorA$p)

}

module = scolor

pheno = colnames(pho6)[x]

modNames = substring(colnames(mergedMEs), 3)

module_column = match(module, modNames)

pheno_column = match(pheno,colnames(pho6))

moduleGenes = moduleColors == module

pdf("019MMvsGS.pdf",width=5,height = 5)

par(mfrow = c(1,1))

verboseScatterplot(abs(geneModuleMembership[moduleGenes, module_column]),

abs(geneTraitCor[moduleGenes, pheno_column]),

xlab = paste("Module Membership in", module, "module"),

ylab = paste("Gene significance for", pheno),

main = paste("Module membership vs. gene significance\n"),

cex.main = 1.2, cex.lab = 1.2, cex.axis = 1.2, col = module)

abline(h=0.8, col = "red")

abline(v=0.8, col = "red")

dev.off()

#

wsc<-which(str_detect(colnames(datKME),scolor))

head(data2[1:4,1:4])

class(data2)

FilterGenes=which(mergedColors==scolor)[abs(GS1)[which(mergedColors==scolor)]>0.8& abs(datKME[moduleGenes,wsc])>0.8]

which(abs(GS1)[which(mergedColors==scolor)]> 0.9)

which(abs(datKME[moduleGenes,wsc])>0.9)

dimnames(data.frame(data2))[[2]][FilterGenes]

trait_hubGenes<-colnames(data2)[FilterGenes]

save(trait_hubGenes,file='trait_hubGenes.Rdata')

save(GS1,file='GS1.Rdata')

write.csv(trait_hubGenes,"hubgene.csv")

########VEEN###########################################################

setwd("H:/HOXA10/HOXA10 分析02//")

library(grid)

library(futile.logger)

library(VennDiagram)

pdf("DEGgene veen.pdf",width=5,height = 5)

venn.plot <- draw.pairwise.venn( area1 = 418, area2 = 341, cross.area = 123,

category = c("RNAi", "OE"), fill = c("dodgerblue","firebrick"), lty = "blank", cex = 3, cat.cex = 2,

cat.pos = c(20, 20), cat.dist = 0.09, cat.just = list(c(2.5, 1), c(0.5, 1)),

ext.pos = 30, ext.dist = -0.05, ext.length = 0.85, ext.line.lwd = 2, ext.line.lty = "dashed" );

grid.draw(venn.plot)

dev.off()

pdf("WGCNA DEG gene veen.pdf",width=5,height = 5)

venn.plot <- draw.pairwise.venn(area1 = 180, area2 = 636, cross.area = 42,

category = c( "WGCNA","DEGs"), fill = c("salmon4","mediumpurple"), lty = "blank", cex = 3, cat.cex = 2,

cat.pos = c(192, 205), cat.dist = 0.09, cat.just = list(c(0.5, 1), c(2, 1)),

ext.pos = 30, ext.dist = -0.05, ext.length = 0.85, ext.line.lwd = 2, ext.line.lty = "dashed" );

grid.draw(venn.plot)

dev.off()

####GO KEGG##############################

degree<-read.csv("degree.csv",header = T)

#

library(ggplot2)

library(clusterProfiler)

library(org.Hs.eg.db)

library(AnnotationDbi)

library(stats4)

library(BiocGenerics)

library(parallel)

df <- bitr(unique(degree$Name), fromType = "SYMBOL",

toType = c( "ENTREZID"),

OrgDb = org.Hs.eg.db)

#

#

library(clusterProfiler)

gene_diff=df$ENTREZID

length(gene_diff)

kk.up<- enrichKEGG(gene = gene_up,

organism = 'hsa',

universe = gene_all)

kk.down <- enrichKEGG(gene = gene_down,

organism = 'hsa',

universe = gene_all)

barplot(kk.down,drop=T,showCategory = 12,title = 'bar_down')

barplot(kk.up,drop=T,showCategory = 12,title='bar_up')

dotplot(kk.down,showCategory = 12,title = 'dot_down')

dotplot(kk.up,showCategory = 12,title='dot_up')

library(org.Hs.eg.db)

ego_CC <- enrichGO(gene = gene_diff,

OrgDb= org.Hs.eg.db,

ont = "CC",

pAdjustMethod = "BH",

readable = T)

ego_BP <- enrichGO(gene = gene_diff,

OrgDb= org.Hs.eg.db,

ont = "BP",

pAdjustMethod = "BH")

ego_MF <- enrichGO(gene = gene_diff,

OrgDb= org.Hs.eg.db,

ont = "MF",

pAdjustMethod = "BH")

barplot(ego_CC, showCategory=12,title="bar_CC")

barplot(ego_BP, showCategory=12,title="bar_BP")

barplot(ego_MF, showCategory=12,title="bar_MF")

dotplot(ego_CC,showCategory = 12,title="dot_CC")

dotplot(ego_BP,showCategory = 12,title="dot_BP")

dotplot(ego_MF,showCategory = 12,title="dot_MF")

save(kk.up,kk.down,ego_BP,ego_CC,ego_MF,file='annotation.Rdata')

barcc<-barplot(ego_CC, showCategory=12,title="bar_CC")

ggsave(file="barcc.png",plot=barcc,width=10,height = 4)

barBP<-barplot(ego_BP, showCategory=12,title="bar_BP")

ggsave(file="barBP.png",plot=barBP,width=10,height = 4)

barMF<-barplot(ego_MF, showCategory=12,title="bar_MF")

ggsave(file="barMF.png",plot=barMF,width=10,height = 4)

dotcc<-dotplot(ego_CC,showCategory = 12,title="dot_CC")

ggsave(file="dotcc.png",plot=dotcc,width=10,height = 4)

dotBP<-dotplot(ego_BP,showCategory = 12,title="dot_BP")

ggsave(file="dotBP.png",plot=dotBP,width=10,height = 4)

dotMF<-dotplot(ego_MF,showCategory = 12,title="dot_MF")

ggsave(file="dotMF.png",plot=dotMF,width=10,height = 4)

#

load("data1.Rdata")

DFgene<-read.csv("degree.csv",header = T)

head(hoxsi2)[1:4,1:4]

data<-hoxsi2[,c(which(colnames(hoxsi2)=="HOXA10"),which(colnames(hoxsi2)%in%DFgene$Name))]

data1<-data

#

library(corrplot)

library(Hmisc)

library(lattice)

library(survival)

library(Formula)

library(ggplot2)

chayi1 <- rcorr(as.matrix(data1),type = "spearman")

write.csv(chayi1$r,"chayir.csv")

write.csv(chayi1$n,"chayin.csv")

write.csv(chayi1$P,"chayip.csv")

pdf("xiangguanxinghox.pdf",width=6,height = 6)

corrplot(chayi1$r, mar=c(3,6,6,3),type="upper", order="hclust", p.mat = chayi1$P, sig.level = 0.05,

insig = "blank",number.cex=0.1,tl.pos = "td",tl.cex = 0.55,tl.col="black")

corrplot(chayi1$r, method = "circle", type = "upper", tl.pos = "lt",tl.cex = 0.55,tl.col="black")

corrplot(chayi1$r, add = TRUE, type = "lower", method = "number", diag = FALSE,

tl.pos = "n", cl.pos = "n",number.cex=0.4)

corrplot(chayi1$r, method = "circle", type = "upper", tl.pos = "lt",tl.cex = 1,tl.col="black", tl.srt=45)

corrplot(chayi1$r, add = TRUE, type = "lower", method = "number", diag = FALSE,

tl.pos = "n", cl.pos = "n",number.cex=1)

dev.off()

####################key genes comparison#####################

load("volcanoHOXOE.Rdata")

load("volcanoHOXRNAI.Rdata")

TTVOE$gene_name<-rownames(TTVOE)

TTVRNAI$gene_name<-rownames(TTVRNAI)

TT<-merge(TTVOE,TTVRNAI,by="gene_name")

TT1<-TT[which(TT$change.x!=TT$change.y),]

table(TT1$change.x)

table(TT1$change.y)

hub<-read.csv("hubgene.csv",header = T)

names(hub)[2]<-"gene_name"

hubtt<-merge(hub,TT1,by="gene_name")

BB<-rbind(TTVOE,TTVRNAI)

BB1<-BB[!duplicated(BB$gene_name),]#

hubbb<-merge(hub,BB1,by="gene_name")

write.csv(hubbb,"hubbb.csv")

SS<-merge(TTVOE, TTVRNAI, by = "gene_name", all = TRUE)

write.csv(SS,"SS.csv")

SS<-read.csv("SS.csv",header = T)

degree<-read.csv("degree.csv",header = T)

SS1<-SS[c(which(SS$gene_name=="HOXA10"),which(SS$gene_name%in%degree$Name)),]

write.csv(SS1,"SS1.csv")

save(SS1,file='SS1.Rdata')

##

load("data1.Rdata")

load("data2.Rdata")

load("SS1.Rdata")

head(hoxsi2)[1:4,1:4]

head(data2)[1:4,1:10]

colnames(data2)[1:100]

keygene<-data2[,which(colnames(data2)%in%SS1$gene_name)]

keygene1<-log2(keygene)

write.csv(keygene1,"keygene1.csv")

keygene1$group[1:5]<-"ZsGreen"

keygene1$group[6:10]<-"HOXA10OE"

keygene1$group[11:15]<-"siNC"

keygene1$group[16:20]<-"siHOXA10"

keygene1$sample<-rownames(keygene1)

keygene2<-keygene1[,c(ncol(keygene1),ncol(keygene1)-1,1:(ncol(keygene1)-2))]

library(cowplot)

library(tidyverse)

library(ggplot2)

library(ggsci)

library(ggpubr)

library(dplyr)

a1<-keygene2[1:10,]

colnames(a1)

mydata<-a1 %>%

gather(key="gene",value="Expression", c(colnames(a1)[3:ncol(a1)])) %>%

dplyr::select(sample,gene,Expression,everything())

head(mydata)

mydata$group = factor(mydata$group, levels=c('ZsGreen','HOXA10OE'))#调换位置

compare_means(Expression ~ group, data = mydata)

#

h<-ggplot(mydata, aes(gene, Expression, fill = group))+

geom_boxplot(alpha=0.7) + expand_limits(y=c(0,12))+

geom_boxplot(aes(color = group))+

stat_compare_means(aes(group = group),method="t.test",label = "p.signif",size=6,label.y = 10)

h1<-h+ theme_bw()+

theme(panel.grid.major = element_blank(),

panel.grid.minor = element_blank())+

scale_fill_manual(values=c("green", "red"))+

scale_color_manual(values = c("black", "black"))+

theme(panel.grid.major = element_blank(),panel.grid.minor = element_blank())+

theme(axis.text.x=element_text(angle=45,hjust=1, vjust=1,size = 10,colour = "black"))

print(h1)

ggsave(h1,filename = 'compare 01.pdf',width= 8,height = 3)

#

a1<-keygene2[11:20,]

colnames(a1)

mydata<-a1 %>%

gather(key="gene",value="Expression", c(colnames(a1)[3:ncol(a1)])) %>%

dplyr::select(sample,gene,Expression,everything())

head(mydata)

mydata$group = factor(mydata$group, levels=c('siNC','siHOXA10'))#调换位置

compare_means(Expression ~ group, data = mydata)

h<-ggplot(mydata, aes(gene, Expression, fill = group))+ #fill填充箱式图颜色

geom_boxplot(alpha=0.7) + expand_limits(y=c(0,12))+

geom_boxplot(aes(color = group))+

stat_compare_means(aes(group = group),method="t.test",label = "p.signif",size=6,label.y = 10)

h1<-h+ theme_bw()+

theme(panel.grid.major = element_blank(),

panel.grid.minor = element_blank())+

scale_fill_manual(values=c("green", "skyblue"))+ #箱式图的颜色

scale_color_manual(values = c("black", "black"))+ #箱式图线的颜色

theme(panel.grid.major = element_blank(),panel.grid.minor = element_blank())+

theme(axis.text.x=element_text(angle=45,

hjust=1, vjust=1,size = 10,colour = "black"))#hjust=1是末端对齐，0.5是中间

print(h1)

ggsave(h1,filename = 'compare RNAiNC01.pdf',width= 7.9,height = 3)

#

library(pheatmap)

library(RColorBrewer)

keygene3<-keygene2[c(6:10,1:5,11:20),]

keygene4<-keygene3[,-c(1,2,7)]

data_matrix1<-t(keygene3[,-c(1,2)])

data_matrix<-t(keygene4)

max(keygene2[,-c(1,2)])

min(keygene2[,-c(1,2)])

bk = c(seq(2,8, length=100))

table(keygene2$group)

rownames(keygene3)

#

annotation_col = data.frame(Group = keygene3$group,

HOXA10 = data_matrix1[which(rownames(data_matrix1)=="HOXA10"),])

ann_colors = list(Group = c(HOXA10OE= "red", ZsGreen = "green", siNC= "lightgreen",siHOXA10= "skyblue"),

HOXA10 = colorRampPalette(c("navy", "white", "firebrick3"))(100))

pheatmap(data_matrix,

breaks=bk,#

legend_breaks=c(2,4,6,8),

show_rownames = T,

show_colnames = F,

cluster_col = F,

cluster_row = T,

border = NA,

cellheight = 22,

cellwidth = 10,

fontsize = 10,

fontsize_row = 10,

fontsize_col = 10,

#display_numbers = p21,

annotation_col = annotation_col,

annotation_colors = ann_colors,

color = colorRampPalette(c("navy", "white", "firebrick3"))(100),

filename = 'pheatmap.pdf',width = 10,height = 8 )

##

#####corrplot##############################################################

ENDO<-read.csv("HOXA10 DDPCR QPCR.csv",header=TRUE)

names(ENDO)

names(ENDO)[1]<-"ΔCt"

names(ENDO)[2]<-"MR(%)"

HOXA10<-ENDO$M

test<-data.frame(dt,HOXA10)

pairs(test)

cor.test(dt,HOXA10)

#

library(PerformanceAnalytics)

library(xts)

library(zoo)

chart.Correlation(ENDO, histogram=TRUE, pch=19)

library(ggplot2)

r1<-cor.test(ENDO$ΔCt,ENDO$`MR(%)`,

alternative = "two.sided",method = "spearman",conf.level = 0.95)

p<-r1$p.value

r<-r1$estimate

par(mar=c(3,6,3,3))

p1<-ggplot(data=ENDO, aes(x=ΔCt, y=`MR(%)`)) +

geom_point(color="#d7191c") +

geom_smooth(method="lm",color="#1a9641") +

geom_text(aes(x=12.5, y=40,label=paste("R","=",signif(r,3),seq=""),hjust=3),color="black")+

theme_bw()+

geom_text(aes(x=12.5, y=40,label=paste("p","=",signif(p,3),seq=""),hjust=5.42,vjust=2),color="black")+

theme(panel.grid.major = element_blank(),panel.grid.minor = element_blank())+

theme(axis.text.x = element_text(size=10,color="black"))+

theme(axis.text.y = element_text(size=10,color="black"))+

theme(text = element_text(size = 15)))

ggsave(file="XIANGGUANXING CT-DD HOXA10.pdf",plot=p1,width=3,height = 3)
